# Sugars and Health: The Science Behind the Hype

**DOI:** 10.3389/fnut.2014.00026

**Published:** 2015-01-07

**Authors:** Mike Gibney

**Affiliations:** ^1^Institute of Food and Health, University College Dublin, Dublin, Ireland

**Keywords:** sugar, high fructose corn syrup, nutritive sweeteners, non nutritive sweeteners, obesity

This book is a most welcome initiative at a time when the nutritional value of sugars is coming under increased scrutiny by writers of popular books and by the media. It is also timely that such a comprehensive review becomes available as the World Health Organization revises its opinion on sugars and health. The book is divided into five sections: an overview, a focus on the global perspectives, on the functional effects of sugars, on sugars and health, and on sugars in chronic disease. In all, 37 authors contribute to 21 chapters, each of which begins with a series of key points.

One of the most important chapters in the first section provides an historical analysis of all nutritional sweeteners but with a special focus on high fructose corn syrup (HFCS), its production, and its composition. It shows that as HFCS was introduced into the US, that sucrose intake fell in parallel to the rise in HFCS over the next 40 years. During this period, obesity rates doubled but total sweetener usage did not show a comparable increase. In this section, Professor Luc Tappy from the University of Lausanne outlines the metabolism of nutritive sweeteners in the body and he concludes in respect to fructose ingestion in humans that 50% of the absorbed fructose is released by the liver as glucose, 25% as lactate, and that 20% is stored as liver glycogen. A minor portion (5%) is secreted into the blood as very low- density lipoproteins. An important chapter in this section examines the evidence that liquids high in nutritive sweeteners might contribute to obesity and the authors conclude: “The epidemiological data, short-term appetite and feeding studies, randomized controlled trials, and mechanistic findings strongly suggest, but do not confirm, that energy-yielding beverage consumption is directly related to risk of weight gain.”

The next section deals with the global perspectives and looks at worldwide trends in nutritive sweeteners intake. The US data are very detailed broken down by age and sex and for all sources of nutritive sweeteners. For the rest of the world, the data are quite limited and to some extent the chapter title is misleading. However, there are some interesting data. For example, in Asia over the years 1963, 1973, 1983, 1993, 2003, per capita sugar intake (gram/capita/day) rose from 11.0, to 16.4, to 21.9, to 30.1 finishing in 2003 at 35.6. In contrast, the corresponding figures for Europe are 82.2, 98.6, 93.2, 87.7, and 93.2. It is thus possible to imagine that sugar might have contributed to weight gain in Asia but in Europe where weight gain soared at US levels, sugar intakes fluctuated very little. It should also be noted that the EU agricultural policy limits the contribution of HFCS to overall sweetener production to just 5%. It is thus difficult to argue for a unique role of HFCS in the US obesity epidemic when that same epidemic happened in the EU with little or no HFCS. This section also contains a chapter looking at randomized trials and prospective cohort studies that examined the obesity-added sugars links. The actual focus of the chapter is on fructose, however, and that rightly allows the author to conclude that added fructose containing sugars do not contribute to adverse health effects relative to other forms of carbohydrate in the diet.

The third section examines functional aspects and there is an excellent chapter, which looks at post-prandial blood levels of hormones, appetite regulating peptides, and standard metabolites in subjects given diets rich in sucrose and HFCS. No significant differences were observed but then, that is what one would expect since the two nutritive sweeteners are so close in nutritional composition. Another important chapter in this section deals with addiction. It concludes that the standard medical classification of addiction (DSM-V criteria) do not support a case for sugar addiction. An alternative approach is the Yale Food Addiction Scale (YFAS) but whereas this tool seems to be associated with certain eating disorders, it does not seem to be linked to obesity.

The penultimate section deals with sugars and health and has a strong emphasis on children. The authors conclude that: “Aside from dental caries, sugars do not appear to be directly related to health issues”. A possible role for sugar-sweetened beverages is not excluded as a contributor to weight gain by the authors where higher sugar intakes lead to higher energy intakes.

These varying opinions within this book reinforce in the mind of the reader that this is a balanced, well-researched, and highly useful publication. Certainly, this book is an essential addition to the library shelves of universities, food companies, and governmental organizations. This review can only look at a few of the many chapters but there is very little in the area of food and health in relation to nutritive sweeteners that is not covered comprehensively in this excellent book.

## Conflict of Interest Statement

The author declares that the research was conducted in the absence of any commercial or financial relationships that could be construed as a potential conflict of interest.

